# First report of chronic invasive fungal rhinosinusitis in a patient with ovarian cancer caused by *Didymella pedeiae* and successful treatment with voriconazole: A case report

**DOI:** 10.18502/cmm.7.1.6244

**Published:** 2021-03

**Authors:** Omid Raiesi, Seyed Jamal Hashemi, Muhammad Ibrahim Getso, Pegah Ardi, Mojtaba Mohammadi Ardehali, Vahid Raissi, Sina Shamsaei, Zeinab Borjian Boroujeni

**Affiliations:** 1 Department of Medical Parasitology and Mycology, Faculty of Public Health, Tehran University of Medical Sciences, Tehran, Iran; 2 Department of Medical Microbiology and Parasitology, College of Health Sciences, Bayero University, PMB 3011, Kano, Nigeria; 3 Department of Otorhinolaryngology, Faculty of Medicine, Tehran University of Medical Sciences, Amiralam Hospital, Tehran, Iran; 4 Department of Medical Parasitology and Mycology, Faculty of Medicine, Iran University of Medical Sciences, Tehran, Iran

**Keywords:** Chronic invasive fungal rhinosinusitis, *Didymella pedeiae*, Iran, *Phoma* species, Voriconazole

## Abstract

**Background and Purpose:**

*Didymella pedeiae* is a dematiaceous fungus that belongs to the Coelomycetes class. While species within this class are known to cause human infection, *D. pedeiae* had previously only been known as phytopathogens and had never been isolated from a human sample.

**Case report:**

A 51-year-old Iranian female patient with ovarian cancer was admitted with unilateral lesions in paranasal sinuses and a five-month history of nasal obstruction, headache, postnasal drainage, swelling on the left side of the face, and orbital pain. Paranasal sinus computerized tomography scan revealed a soft tissue mass that filled the left nasal cavity, ethmoid, sphenoid, and frontal sinuses with more involvement in the maxillary and ethmoid sinuses. Antifungal treatment was simultaneously initiated with itraconazole+prednisolone 15 mg/day, and levofloxacin. Due to poor clinical response, IV voriconazole and amphotericin B were added to the treatment as well. The patient recovered completely after 10 weeks of therapy.

**Conclusion:**

Here, we report the first case of human *D. pedeiae* infection in a patient with ovarian cancer.

## Introduction

Fungal rhinosinusitis (FRS) involves a number of sinonasal disorders caused by fungal agents and runs a variable clinical course, histopathologic findings, and disease outcomes. The FRS can be categorized into invasive and noninvasive diseases based on the degree to which the mucosal layer is invaded by the fungi. Non-invasive FRS includes those caused by saprophytic fungi, the fungal ball, and allergic FRS. Furthermore, the invasive FRS is classified into acute and chronic invasive FRS [ 1 ]. 

Chronic invasive fungal rhinosinusitis (CIFRS) is a rare form of fungal rhinosinusitis, which follows a prolonged clinical course with slow disease development, typically more than 12 weeks. The disease shows radiologic and histopathological evidence of hyphae in sinus mucosa, submucosa, blood vessels, or bone [ 2 ]. 

*Phoma* spp. (synonym *Didymella*) are dematiaceous fungi that belong to the class Coelomycetes, order Sphaeropsidales, and family Dematiaceous with over 2000 species described. They are phytopathogens and ubiquitous; commonly found in plants, soil, water sources, and organic matter. In the existing literature, few cases of human and animal phaeohyphomycosis caused by *Phoma* spp. have been described [ 3 ]. In this study, we report the first case of CIFRS due to *Didymella pedeiae* in a patient with ovarian cancer.

## Case report

A 51-year-old Iranian female patient with ovarian cancer was admitted with unilateral lesions in paranasal sinuses and five-month history of nasal obstruction, headache, postnasal drainage, swelling on the left side of the face, and orbital pain. At the time of admission, the vital signs of the patient were as follows: temperature: 37.8°C, blood pressure: 115/65 mm Hg, heart rate: 85 beats/min, respiratory rate: 32 breaths/min, and O_2_ saturation: 90% in ambient air.

The patient had no history of any immune-suppressing condition and no history of steroid intake prior to the current event. However, during the course of her cancer treatment, she received induction chemotherapy with platinum agents (cisplatin, carboplatin, and paclitaxel). Moreover, she simultaneously received prednisolone 15 mg/day, levofloxacin, and itraconazole for empirical treatment of rhinosinusitis, which did not improve, raising suspicion of fungal rhinosinusitis.

The patient was subjected to diagnostic nasal endoscopy and CT scan of paranasal sinuses (PNS). Results of the PNS CT scan revealed a soft tissue mass that filled the left nasal cavity, ethmoid, sphenoid, and frontal sinuses with much involvement of the maxillary and ethmoid sinuses. Bone destruction and chronic sinus osteomyelitis were also observed. The endoscopic examination revealed a polypoid mass in the left nasal cavity with intense purulent secretion; consequently, surgery was performed to completely remove the lesions. The brown-green substance was fully resected from all sinuses through endoscopic sinus surgery. Biopsied tissues were also sent separately, submerged both in 0.9% sterile saline and 10% formalin, to the mycology and pathology department. 

The wet mount (KOH 10%) preparation of the sample showed septated, branched, and pigmented fungal hyphae suggestive of *Phoma* spp. 

**Figure 1:**
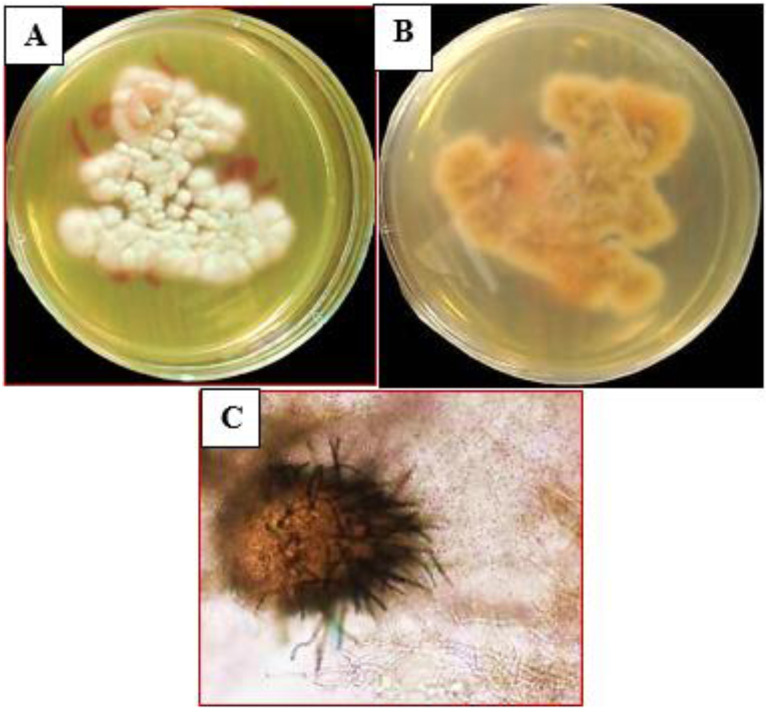
*Phoma pedeiae*: (a) colony on 2% dextrose Sabouraud Agar with 0.05 g/L chloramphenicol, (b) yellowish-brown reverse of colony, (c) pycnidia typical of the genus *Phoma*

Fungal culture was done two days ‎after surgical debridement and was positive for *Phoma* spp. To cultivate the fungal agent, the sample was inoculated onto the brain-heart infusion agar (BHIA), Sabouraud’s dexterous agar (SDA), and Sabouraud’s dextrose agar containing chloramphenicol (SC), under sterile conditions. The SDA and SC culture media were incubated at 25 °C and BHIA at 35 °C. The culture growth was powdery to velvety, white-cinnamon in color with yellowish-brown reverse attaining a diameter of six cm within one week. After three weeks of incubation at 25 °C, partially submerged pycnidia were microscopically seen (Figure 1).

**Figure 2:**
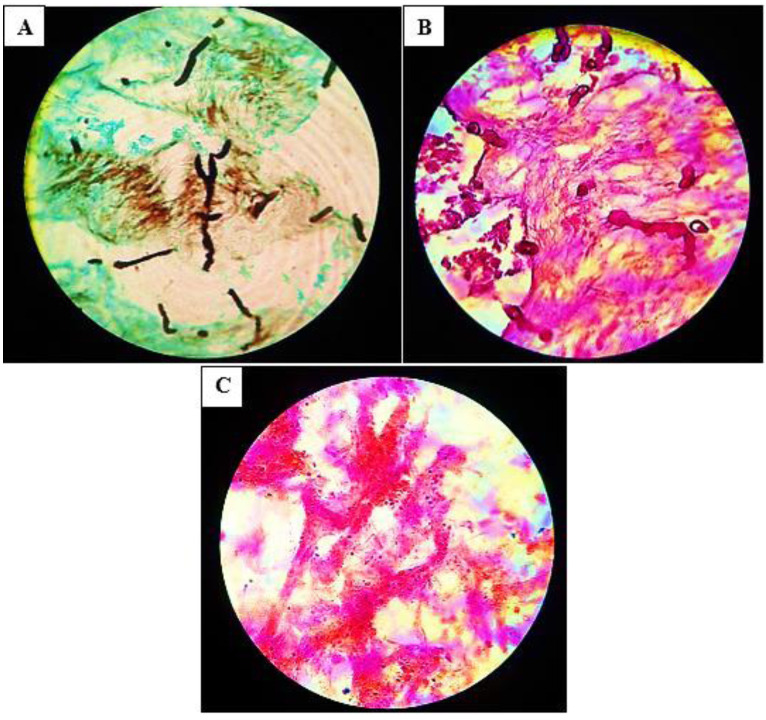
Gomori Methenamine-Silver and periodic acid-Schiff stain showing an invasion of fungal forms into the submucosa (a, b), and Haematoxylin and Eosin stain showing surrounding chronic inflammation and fibrosis (c)

Histologically, the slides were stained with Haematoxylin and Eosin (H&E), Periodic acid-Schiff (PAS), and Gomori Methenamine-Silver (GMS). One week after debridement, the results of H&E, PAS, and GMS-stained were available. Investigation of H&E, PAS, and GMS-stained sections showed invasion of fungal hyphae into submucosa surrounded with evidence of chronic inflammation and fibrosis in the lesion (Figure 2).

The fungus grown on Sabouraud dexterous agar (SDA) was then transferred to a 2 ml Eppendorf tube containing 400 ml TEX buffer (Tris 1.2 % w/v, Na-EDTA 0.38 % w/v, pH 9.0) with glass beads (Sigma) and homogenized by MO-BIO vortexing for 15 min. The DNA was extracted and subjected to molecular identification. The obtained sequences were analyzed in the GenBank database using NCBI BLAST search tools (https://blast.ncbi.nlm.nih.gov/Blast.cgi) and the fungal identities were determined through comparison with the highest matches in DNA databases [ 4 ]. The nearest neighbor to our isolate within the ITS BLAST in GenBank was *Phoma pedeiae*, with 99 % similarity. The sequence generated and analyzed during the current study is available in the GenBank under the code MT755856.1.

Susceptibility of the *Didymella pedeiae* isolates to amphotericin B, voriconazole, itraconazole, and fluconazole was determined using the Clinical and Laboratory Standards Institute M38-A2 standard method [ 5 ]. Results of the in vitro susceptibility tests revealed that the isolate was susceptible to amphotericin B, and voriconazole. Antifungal treatment was immediately initiated with IV voriconazole (400 mg/day for the first day and then 200 mg bid/po) and amphotericin B (1–1.5 mg/kg/day) until the total dose of amphotericin B reached 2 grams. The patient recovered completely after 10 weeks of therapy. 

## Ethical Considerations

The work was approved by the Research Council of Tehran University of Medical Sciences with the project ethic number IR.TUMS.SPH.REC.1397.247. Written informed consent for publication of this case report was obtained from the patient. The sequence generated and analyzed during the current study is available in the GenBank under the code MT755856.1.

## Discussion

Based on the available literature, human *Phoma* spp. infections are extremely rare. For the first time, *Phoma hibernica* was isolated from a lesion on the left leg of a young girl in Ontario, Canada [ 3 ]. In 1987, Baker et al. reported the first case of subcutaneous phaeohy-phomycosis of the foot caused by *Phoma minutella* in a 75-year-old diabetic farmer from the Dominican Republic [ 6 ]. In 2016, Hernández reported the first case of chromoblastomycosis due to *phoma insulana* [ 7 ]. A rare case of keratitis caused by *Phoma gardeniaea* was reported by Miyakubo et al. in a 66-year-old man with a history of bronchial asthma and duodenal ulcer [ 8 ]. In another study, *Phoma* spp. was isolated from keratitis in a 72-year-old man [ 9 ]. More, recent studies have also reported cases of *Phoma* infections in other parts of the human body. For instance, *Phoma cava* from the left ear, subcutaneous, and hand [ 10 , 11 ]; *Phoma sorghina* from face, neck, and hands [ 12 ]; *Phoma exigua* from lung [ 13 ]; *Phoma macrostoma* from onychomycosis[ 14 ]; *Phoma herbarum* from nail and toe [ 15 ]; and *Phoma* spp. from corneal infection [ 16 ].

Management of CIFRS involves surgical removal of the affected tissue, antifungal treatment, and supportive therapy to help reverse the predisposing factors. Different therapeutic options have been used in clinical cases, most of which had satisfactory outcomes. In the studies performed by Salehi (2019) [ 17 ], Roehm (2012) [ 18 ], and Errera (2008) [ 7 ], voriconazole and amphotericin B showed the best activity against *Phoma* isolates. These last findings are similar to those observed for *P. pedeiae*. Due to the rare reports of cases caused by *Phoma* species and the insufficient knowledge on specific treatment modalities of these patients, there is a need for further studies. The choice of treatment protocol and duration of treatment can vary with the overall clinical condition of the patients and the type of *Phoma* spp.

## Conclusion

Although this fungus is ubiquitous in the environment, its poor invasive capacity could be assumed to be a factor linked to its low incidence in deep infection. However, immunosuppression should be considered as a possible additional factor for *phoma* spp infection, especially among patients from rural settings. Therefore, accurate characterization of rare fungal pathogens from clinical material of immunocompromised patients is of medical and epidemiological significance.

## Authors’ contribution

S.J.H. conduction of the study, O.R., M.I.G. and Z.B. morphological and molecular analysis of the isolated strain. M.A., P.A. and V.R. collection of clinical data. O.R. histopathological examination. O.R., M.I.G., and S.J.H. design of the study and preparation of the final draft of the manuscript. All authors read and approved the final manuscript.

## Financial disclosure

This study was supported by the Research Council of Tehran University of Medical Science and the project funding number is IR.TUMS.SPH.REC.1397.247.
